# Finance Administration

**Published:** 1894-07-07

**Authors:** 


### The Institutional Workshop.

### HOSPITAL FINANCE.

### IX.—FINANCIAL ADMINISTRATION.

Hospital administration, so far as it touches finance, presents the widest variations from the most carefully organised system of cautious expenditure to that familiar method known as "happy-go-lucky." To America and Sweden we must look for examples of the former, while for the latter no very long journey or arduous search need be undertaken. A perfect system of finance administration must render debt an impossibility by a prudent forecast of income, and the adjustment of expenditure to fall well within it. In Sweden the directors of the State hospital at Christiania annually submit their budget to Parliament, and any deficiency is made good by a Parliamentary vote; the income being thus elastic, the expenditure is not hampered, though in practice it often happens that no vote is required. In some hospitals in the United States, where the voluntary system is in full force, an equally business-like arrangement is common. "In the hospital of the University of Pennsylvania, the Board of Governors, from time to time, usually about twice a year, after reviewing the financial situation, issue an order fixing the maximum number of free beds, which may be filled by the superintendent, subject, however, to the conditions that cases of accidents requiring immediate hospital care are to be received even if the prescribed number of free beds is full. When, however, by reason of the admission of such special accident cases, the number of free patients has been made to exceed the limit fixed by the Board of Governors, no more free patients can be admitted until by discharge or death the number has been reduced below this limit. The beds in excess of this limit then become available for pay patients, at rates just equivalent to the extra expense they cause. In a community of business men the fact that the managers of a hospital refuse to undertake more than the income justifies, and decline to incur indebtedness which there is no definite prospect of meeting, tends to create confidence, and a willingness to give substantial aid when the need for such aid is demonstrated."*

It is strange that these principles of common sense and business prudence should have as yet gained so little ground among English charitable institutions, managed and supported as they are by business men who would shudder at the thought of ordering their private affairs on lines of incessant debt and difficulty. An examination of the published accounts of the London hospitals for the year 1892, reveals the fact that in 29 cases, nearly half the total number, the expenditure was in excess of the income, and this, in some instances, to an extent of several thousand pounds. Freedom from debt, always reckoned in the case of private persons the first recommendation towards assistance, will assuredly, as public wisdom increases in the matter of giving, prove a more efficacious plea for an institution than an advertisement of many thousands of liabilities which there is no prospect of meeting.

* "Hospitals of the World," page 712. H. C. Burdett.

As regards the central authority for the regulation of expenditure, hospitals fall into two great classes, the voluntary and the State controlled. In the former class are the hospitals of the United Kingdom, certain of the Colonies, and the United States of America; in the latter, those of most other countries in the world. It will be instructive to compare some of the most striking features of financial organisation in other countries before proceeding to examine the traditional methods of our own hospitals. Speaking generally, the great distinction will be found to be the enormous amount of unpaid work carried through on the voluntary system, no part of which is so amazing to foreigners as the readiness with which men, weighted with public and private business, surrender valuable hours for the transaction of affairs, not even remotely connected with their own interests.

Perhaps no country has so perfect a genius for organisation as France. By the law of 1851, supplemented by others in 1873 and 1879, all the hospitals in the Republic have been brought under one uniform administration, and the regulations are so complete and exhaustive that it would seem a matter of impossibility for any French hospital to go very far wrong. The administrative committee of each hospital decides on all ordinary questions of finance, regulates the budgets, accounts, and all the receipts and expenses, including extraordinary expenses within the limits of £120. The administrative committee is, however, under the direct control of the municipal council of its commune, which is empowered to examine the hospital budgets, receive donations and bequests on their behalf, and give authorisation, when necessary, for the incurring of extraordinary expenses above the specified limits. The line of communication with the State is completed by the intervention of the prefect of each department in the control of hospital property.

In the Belgian hospitals the contrary principle of decentralisation is in practice. Each commune is responsible for the financing of its own charitable institutions, and the work is done by a Commission (commonly of five members), appointed by the communal council. A director, who is responsible solely to the Commission, is placed in charge of each hospital, and the Government has no power of interference except circuitously through the communal council.

In Germany, the Department of the Interior in every State directs all poor and sick relief, and the institutions connected therewith. In certain provinces these rights belong to a communal council with corporation rights. In each case the local authority is subject to Imperil control through the medium of the Ministry of Public Worship, Instruction, and Medicine. An Imperial Health Office was founded in 1876 to support the Imperial Chancellor in superintending the measures of the medical and sanitary police.

Since the reorganisation of charitable foundations in Italy in 1890, the hospitals have been uniformly placed under the control of councils of administration elected partly by the communal, partly by the provincial councils. No person in any way connected with hospitals or related to the medical officers is qualified to sit on these councils. They are supreme in all matters concerning the hospital administration and management. They appoint and dismiss the directors and other salaried officials, and have complete control over all financial matters. The dangers with which such a system is beset among a people almost entirely devoid of any training in the administration of public funds, are too obvious to need pointing out.

In the United States of America the voluntary hospitals are all incorporated under the legislative authority of the State in which they are situated. In most of the States a charities board exercises a certain amount of control over the management, but in financial matters they are all practically independent.

In the voluntary hospitals in England the principal subscribers are theoretically responsible for the management, and are consequently named governors. The theory that those who give the money should control its expenditure has much to recommend it, though as a matter of fact very few people retain the smallest sense of responsibility with regard to their money after it has once passed out of their hands. At the annual meeting of the governors of the hospital a committee of management, commonly of thirty-one persons, is chosen to transact all necessary business, and from this committee members are elected to serve on finance and house committees who meet weekly. The finance committee presents a report once a month to the board of management, which in turn submits a yearly report to the governors. The weak point in the system is the apathy displayed by the general body of governors, to whom the management is nominally responsible, an apathy proceeding partly from dislike to interfering in matters beyond their own sphere, partly to confidenee (generally well founded) in the zeal and ability of those at the head of affairs. There can, however, be little doubt, that most hospital abuses can be traced to the fact that the supreme authority and point of appeal in financial and other matters lies not in one definite body, directly and personally responsible for the well-being of the whole institution, but among a crowd of well-wishers, possessed of no special knowledge, and hence alternately actuated by overconfidence and panic.

